# The Gene-Gene Interaction of INSIG-SCAP-SREBP Pathway on the Risk of Obesity in Chinese Children

**DOI:** 10.1155/2014/538564

**Published:** 2014-06-17

**Authors:** Fang-Hong Liu, Jie-Yun Song, Xiao-Rui Shang, Xiang-Rui Meng, Jun Ma, Hai-Jun Wang

**Affiliations:** School of Public Health, Institute of Child and Adolescent Health, Peking University, No. 38 Xueyuan Road, Haidian District, Beijing 100191, China

## Abstract

*Background.* Childhood obesity has become a global public health problem in recent years. This study aimed to explore the association of genetic variants in INSIG-SCAP-SREBP pathway with obesity in Chinese children. *Methods.* A case-control study was conducted, including 705 obese cases and 1,325 nonobese controls. We genotyped 15 single nucleotide polymorphisms (SNPs) of five genes in INSIG-SCAP-SREBP pathway, including insulin induced gene 1 (*INSIG1*), insulin induced gene 2 (*INSIG2*), SREBP cleavage-activating protein gene (*SCAP*), sterol regulatory element binding protein gene 1 (*SREBP1*), and sterol regulatory element binding protein gene 2 (*SREBP2*). We used generalized multifactor dimensionality reduction (GMDR) and logistic regression to investigate gene-gene interactions. *Results.* Single polymorphism analyses showed that *SCAP* rs12487736 and rs12490383 were nominally associated with obesity. We identified a 3-locus interaction on obesity in GMDR analyses (*P* = 0.001), involving 3 genetic variants of *INSIG2*,* SCAP,* and* SREBP2*. The individuals in high-risk group of the 3-locus combinations had a 79.9% increased risk of obesity compared with those in low-risk group (OR = 1.799, 95% CI: 1.475–2.193, *P* = 6.61 × 10^−9^). *Conclusion.* We identified interaction of three genes in INSIG-SCAP-SREBP pathway on risk of obesity, revealing that these genes affect obesity more likely through a complex interaction pattern than single gene effect.

## 1. Introduction

Obesity is a common disease, which is spreading quickly all over the world. In some developing countries, the prevalence of obesity is rising faster than the historical experience of developed countries [1]. Experts expected that the pandemic will continue to spread for the foreseeable future [2]. What is more, childhood obesity has become a global public health crisis [3].

Genetic factors are associated with pathogenesis of obesity. For obesity is associated with lipid metabolism, the genes related to lipid metabolism are candidate genes for obesity. Proteins from the INSIG-SCAP-SREBP pathway include insulin induced gene proteins (INSIGs), SREBP cleavage-activating protein (SCAP), and sterol regulatory element binding proteins (SREBPs). The inactive precursors of SREBPs are adhered to the endoplasmic reticulum (ER) and their activation requires cleavage by two proteases in the Golgi apparatus [4]. Another two proteins, SCAP and INSIGs, including INSIG1 and INSIG2, are critical for the transportation of SREBPs from ER to Golgi apparatus [5]. SCAP can combine with SREBPs, forming SCAP-SREBPs complex [6]. If the complex combines with INSIGs, the transportation of SREBPs from ER to the Golgi apparatus will be blocked, and finally lipid synthesis will be affected [7]. INSIG1 and INSIG2 mediate feedback control of lipid synthesis by sterol-dependent binding to SCAP [8, 9]. The activation mechanism of SREBPs related to SCAP and INSIGs has been demonstrated [8–10]. In a word, the INSIG-SCAP-SREBP pathway plays a crucial role in feedback regulation of lipid metabolism and may be involved in obesity development.

Among the INSIG-SCAP-SREBP pathway genes,* INSIG2* has been the most frequently studied gene in obesity researches. Herbert et al. [11] discovered that a single nucleotide polymorphism (SNP) rs7566605 in upstream of* INSIG2* was related to obesity. So far, the relationship between rs7566605 and obesity has been reported in different ethnic groups [12–16]. The* INSIG2* rs9308762 polymorphism was reported to be associated with body mass index (BMI) and abdominal circumference in Samoans [17]. The* INSIG2* rs10185316 polymorphism was reported to be associated with weight change in an intervention study [18].

There were a few studies that focused on the relationship between the* INSIG1*,* SCAP* genes and obesity or plasma lipids. Hellard et al. [19] found that* INSIG1* rs13223383 and* SCAP* rs12490383 were related to BMI change in Germans. Two studies, respectively, reported that* INSIG1* rs2721 [20] and* SCAP* rs12487736 [21] were associated with triglyceride. For* SREBP1* and* SREBP2*, most studies focused on their association with plasma lipids [7, 22–25]. Only a few studies described the relationship between* SREBPs* and obesity or BMI [26, 27]. Eberlé et al. [26] reported that a haplotype including three SNPs of* SREBP1* was associated with obesity. Another study in Danish population detected that* SREBP1* rs2297508 was associated with BMI [27].

The previous studies on the INSIG-SCAP-SREBP pathway genes were conducted mostly in adults but very few in children and adolescents. Zavattari et al. [28] conducted a study in obese children and adolescents and suggested that the* INSIG2* rs7566605 may play a role in metabolic complications related to obesity. An intervention study in overweight children showed that rs7566605 had effects on weight change [29]. Our study group had reported that rs7566605 may be associated with severe obesity in Chinese children [16].

We hypothesized that the genetic variants in INSIG-SCAP-SREBP pathway are associated with obesity interactively. Therefore, we conducted a case-control study in Chinese children to investigate the relationship between the genetic variants in INSIG-SCAP-SREBP pathway and obesity.

## 2. Materials and Methods

### 2.1. Subjects

We conducted the case-control study in 2030 subjects of two independent study groups, including 705 obese cases and 1,325 nonobese controls recruited from the urban regions of Beijing, China. The first study group came from the study on adolescent lipids, insulin resistance, and candidate genes (ALIR). The second study group was from the Comprehensive Prevention project for Overweight and Obese Adolescents (CPOOA) with physical exercise and healthy nutrition as instruments. All obese individuals in the selected schools were recruited with their voluntary participation. The method of cluster sampling was adopted to recruit nonobese subjects from some classes of each grade in the same schools. The ALIR subjects were ascertained from adolescents aged 14–17 years in nine middle schools of Dongcheng District of Beijing, including 386 obese adolescents and 551 nonobese adolescents. The CPOOA subjects were recruited from children and adolescents aged 7–18 years in five elementary and middle schools of the Haidian District of Beijing, comprising 319 obese children and adolescents and 774 nonobese children and adolescents. The ascertainment strategies for the two study groups have been described in detail previously [16, 30]. We used the uniform BMI percentile criteria for obese and nonobese children, which were determined in a representative Chinese population [31]. According to the criteria, the children and adolescents with an age- and gender-specific BMI ≥95th percentile are defined as obese, whereas those with a BMI between 15th and 95th percentile are nonobese. The individuals with any cardiovascular or metabolic disease were excluded. Anthropometric measurements, including height and weight, were determined according to standard protocols.

Two studies were approved by the Ethic committee of Peking University Health Science Center. Written informed consent was provided by all participants and, in the case of minors, their parents.

### 2.2. SNPs Selection and Genotyping

Firstly, we searched the SNPs of these five genes that had been reported to be positively associated with obesity or obesity-related phenotypes by previous literatures. With the assumed effect size of 1.3 for an allele frequency ≥0.14, the power for detecting positive association was greater than 0.80. So we selected only SNPs with minor allele frequencies (MAF) ≥0.14 in Chinese individuals in the HapMap database (http://hapmap.ncbi.nlm.nih.gov/). In this way, we selected 10 SNPs that have been shown to significantly associate with obesity or obesity-related phenotypes. Secondly, based on the genotype data downloaded from CHB database of Hapmap, we used Haploview v4.2 to select 5 tagSNPs with MAF higher than 0.14 and having no strong LD with the above SNPs selected from literature (*r* ^2^ < 0.80). In total, 15 SNPs of INSIG-SCAP-SREBP pathway were included in our study. Detailed information about the selected SNPs was shown in Table  1 in Supplementary (see Supplementary Materials available online at http://dx.doi.org/10.1155/2014/538564).

Genomic DNA was extracted from blood leukocytes by phenol-chloroform extraction method. Polymerase chain reaction with subsequent restriction fragment length polymorphism (PCR-RFLP) assay was performed for genotyping the* INSIG2* polymorphisms rs7566605, rs13428113, and rs9308762. The single polymorphism analyses of rs7566605 and rs13428113 had been previously published [16, 32], which were included in the current study for analyzing gene-gene interaction. The other 12 SNPs' genotyping was carried out with iPLEX assays (Sequenom, San Diego, CA, USA), which were designed by the Assay Design Suite 1.0 online software. The assay details of these 15 SNPs are available from the authors upon request. A multiplex polymerase chain reaction was performed, and unincorporated double stranded nucleotide triphosphate bases were dephosphorylated with shrimp alkaline phosphatase followed by primer extension. The purified primer extension reaction was spotted on to a 384-element silicon chip (SpectroCHIP, Sequenom) and analyzed in the Matrix assisted laser desorption ionization time of flight mass spectrometry (MALDI-TOF systems, Sequenom). The genotyping results were processed with Typer 4.0 software (Sequenom). All the experiments were done by investigators who were blind to the phenotypes. The call rates of 15 SNPs were more than 99.0% (see Supplementary Table  1).

### 2.3. Statistical Analyses

The genotype data was tested for deviation from Hardy-Weinberg equilibrium. The differences in general characteristics between obese and nonobese subjects were tested with Pearson Chi-square (categorical variables) or* t*-test (continuous variables). The logistic regression was used to examine the effect of each SNP on obesity and subsequently 100,000 times permutation was performed. Permutation tests can give the optimal exact threshold and are considered the gold standard in multiple testing adjustments for genetic association studies [33].

The gene-gene interaction on obesity was examined by using the general multifactor dimensionality reduction (GMDR) program [34]. GMDR reduces high-dimensional genetic data to a single dimension, explores interaction model through cross validation, and calculates score-based statistics of each subject using maximum likelihood estimates to classify subjects into two different groups (either high-risk or low-risk group). Additionally, 1,000 permutations were performed to get a permutated *P* value of these models. Subsequently, logistic regression was used to confirm the best model of GMDR analyses. All the above analyses were performed under the additive model adjusted for sex, age, age square, and study population. To remove the possibility of spurious associations due to missing genotypes, we only included 2004 samples having genotypes of all SNPs in the GMDR analyses, including 693 obese individuals and 1311 nonobese individuals.

The criterion for statistical significance was set at *P* < 0.05. Pearson Chi-square,* t*-test, logistic regression, and linear regression were performed with SPSS 18.0 software (SPSS Inc., Chicago, USA). *P* value based on 100,000 times permutations was calculated by using PLINK 1.07 software (Massachusetts General Hospital, Boston, MA) [35]. Pairwise linkage disequilibrium (LD) among genetic variants in every gene was estimated using Haploview 4.2 (http://www.broad.mit.edu/mpg/haploview/). The gene-gene interaction analysis was conducted by using GMDR software (beta version 0.7, University of Virginia, Charlottesville, VA). Power calculations were performed using Quanto software (University of Southern California, Los Angeles, CA).

## 3. Results

### 3.1. General Characteristics of the Study Populations

The general characteristics of the study groups were shown in Table 1. Except for age, all the other characteristics were significantly different between the obese and nonobese groups (all *P* < 0.001). Compared with nonobese group, the obese group had more male individuals and higher BMI.

### 3.2. Single Polymorphism Analyses

The distributions of genotypes for the 15 SNPs in INSIG-SCAP-SREBP pathway were shown in Supplementary Table  1. All the polymorphisms were in Hardy-Weinberg equilibrium among the study population (all *P* > 0.05). With linear regression, we did not find that any of the 15 SNPs was associated with BMI (data not shown).

The results of association studies between 15 SNPs and obesity were displayed in Table 2. The* SCAP* rs12487736 and rs12490383 were nominally significantly associated with obesity after adjustment for sex, age, age square, and study population (*P* = 0.039 and 0.026, resp.). The odds ratios (ORs) of rs12487736 and rs12490383 for obesity were 1.15 (95% CI: 1.01–1.32) and 1.17 (95% CI: 1.02–1.33), respectively. After conducting 100,000 permutations, the associations were no longer statistically significant.

### 3.3. Gene-Gene Interaction Analyses

**Figure 1:**
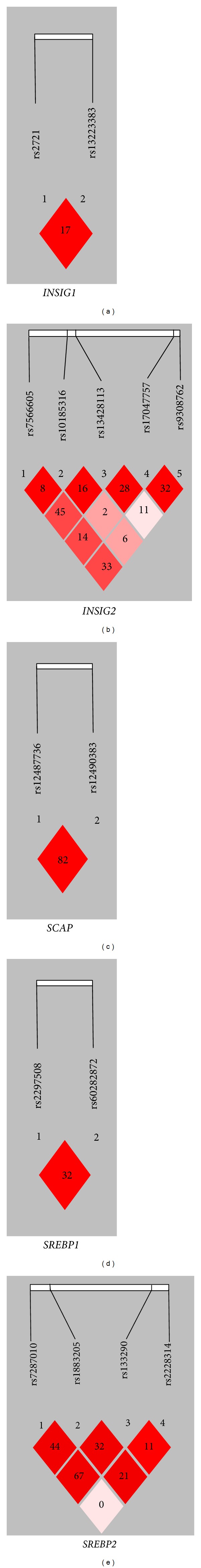
Pairwise linkage disequilibrium (LD) among variants in every gene. Numbers in squares designate the degree of LD (*r* ^2^) between any two markers. LD was estimated using Haploview version 4.2 (http://www.broad.mit.edu/mpg/haploview/).

Pairwise linkage disequilibrium (LD) among genetic variants in every gene was shown in Figure 1. We only found that the rs12487736 and rs12490383 of* SCAP* were in strong linkage disequilibrium (*r* ^2^ = 0.82) and included one of them (rs12487736) in gene-gene interaction analyses. We found a 3-locus model involving 3 genes of INSIG-SCAP-SREBP pathway (*INSIG2* rs9308762,* SCAP* rs12487736, and* SREBP2* rs1883205) had significant gene-gene interaction on obesity (permutated *P* = 0.001). The testing accuracy was 54.95%.

**Figure 2:**
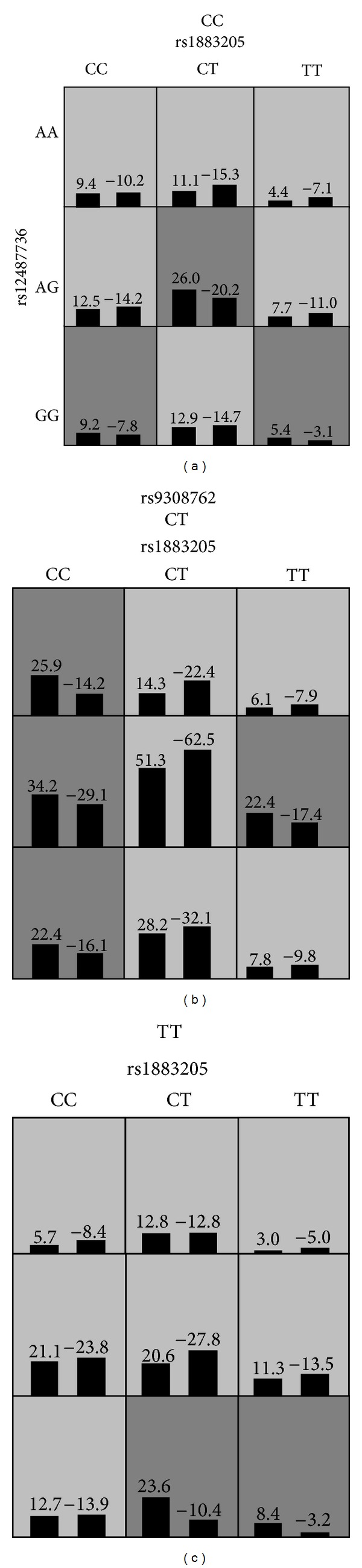
The best model in GMDR analyses was composed of rs9308762, rs12487736, and rs1883205. In each cell of genotype combination, the left bar represents a positive score and the right bar a negative score. High-risk combinations for obesity are shaded dark grey, while low-risk combinations are shaded light grey.

As demonstrated in Figure 2, there were nine genotype combinations of rs9308762/rs12487736/rs1883205 (CC/GG/CC, CC/AG/CT, CC/GG/TT, CT/AA/CC, CT/AG/CC, CT/GG/CC, CT/AG/TT, TT/GG/CT, and TT/GG/TT) having high risk of obesity susceptibility. The frequency differences of 9 high-risk combinations between obese and nonobese children were listed in Table 3. In total, 41.13% obese children and 28.60% nonobese children had high-risk combinations. According to the percentage ratio of obese to nonobese, three genotype combinations (TT/GG/TT, TT/GG/CT, and CT/AA/CC) especially showed high risk for obesity.

Subsequently, we performed logistic regression to evaluate the 3-locus interaction model. The 3-locus genotypes were classified into high-risk or low-risk. Compared with the wild-type genotypes (CC/AA/CC) of 3-locus (rs9308762/rs12487736/rs1883205), the OR values of low-risk genotypes for obesity was lower than 1, that of high-risk genotypes higher than 1 (see Supplementary Table  2). But the *P* values were not significant for the limited sample size in each subgroup. Therefore, we combined 18 low-risk genotypes and 9 high-risk genotypes, respectively, to perform final logistic regression. The result showed that the individuals in high-risk group had a 79.9% increased risk of obesity compared with those in low-risk group with adjustment for sex, age, age square, and study population (OR = 1.799, 95% CI: 1.475–2.193; *P* = 6.61 × 10^−9^).

## 4. Discussion

The novel finding of the present study is gene-gene interaction of the INSIG-SCAP-SREBP pathway on the risk of obesity. Additionally, we found that two* SCAP* SNPs (rs12487736 and rs12490383) had association with obesity (nominal *P* value <0.05).

The GMDR, based on the MDR, is proved to be a useful statistical tool to detect gene-gene interaction [36]. Compared to MDR, GMDR permits the adjustment of discrete and quantitative covariates and is applicable to both dichotomous and continuous data. In our study, the results of GMDR revealed that* INSIG2* rs9308762,* SCAP* rs12487736, and* SREBP2* rs1883205 of INSIG-SCAP-SREBP pathway have gene-gene interaction on obesity. After analyzing frequency differences of genotype combinations of the 3 SNPs between obese and nonobese children, we found that there were 9 high-risk combinations having higher frequency in obese group than that in controls, especially TT/GG/TT, TT/GG/CT, and CT/AA/CC genotypes. The INSIG-SCAP-SREBP pathway plays a crucial role in feedback regulating lipid synthesis. Components of the pathway may not serve as independent factors, because one component usually needs to contact with others in the pathway to complete some biological function [37]. SREBP2 is important to sterol regulation and it is synthesized and located on the endoplasmic reticulum (ER) membrane in their precursor form [38]. The activation of SREBP2 needs INSIGs and SCAP to transfer it to Golgi apparatus [5]. Imbalance of sterol regulation homeostasis could result in some metabolic diseases, such as obesity [39]. Therefore, it is quite important to view these factors as a whole when considering their influence on obesity development. Previous studies showed that genetic interactions can be quite common under biological phenotypes [39, 40]. A few studies investigated interaction of genes in INSIG-SCAP-SREBP pathway on diseases. Smith et al. [20] found the interaction of* INSIG1* rs2721 and* INSIG2* rs7566605 on serum triglyceride levels. A study of coronary heart disease (CHD) in Chinese individuals reported that* INSIG1* and* INSIG2* had gene-gene interaction on risk of CHD occurrence [7]. Recently, interaction of* INSIG1* and* INSIG2* on antipsychotic induced metabolic syndrome was also reported [41].

Although in previous studies* INSIG2* polymorphism rs9308762 had been reported to be associated with obesity or obesity-related traits [17, 42, 43], rs9308762 did not show any association with obesity in our single polymorphism analyses. The* SREBP2* tagSNP rs1883205 also did not show any association with obesity in our single polymorphism analyses. However, when we conducted a GMDR analysis, we found the significant interaction of the three genes (*INSIG2* rs9308762,* SCAP* rs12487736, and* SREBP2* rs1883205) on obesity. A possible reason for the difference between our single polymorphism analyses and GMDR results may be that the single SNP of 3 genes has minor effect on obesity, but they may affect each other in obesity development, which is known as epistasis. The 3 SNPs are involved in INSIG-SCAP-SREBP pathway, so that the interaction may have a functional basis. The gene-gene interaction could provide possible clues for obesity pathogenesis, and further studies evaluating functional epistasis should be performed [44].

Previously Fiegenbaum et al. [21] demonstrated the association of* SCAP* 2386A>G (rs12487736) with lipid metabolism. Lipid metabolism is closely related to obesity development. Hellard et al. [19] reported that the* SCAP* rs12490383 was related to BMI change in schizophrenics treated with antipsychotics, indicating rs12490383 may be susceptive to obesity development. The present study detected that* SCAP* rs12487736 and rs12490383 had nominal association with childhood obesity, which showed similar tendency with previous studies. Children with 2386G allele had higher risk to become obese in our study population. With its biological function and results of genetic epidemiology researches, we could seriously consider the role of* SCAP* in obesity development.

The strength of the study was that we investigated 15 SNPs of five genes in the INSIG-SCAP-SREBP pathway and got more comprehensive information about the effect of the pathway on obesity. Our study was conducted in Chinese children. Compared with adults, children have higher BMI or obesity heritability and most obese children have simple obesity without complications, which help to identify the effects of the gene pathway on obesity.

However, there were a few limitations in the present study. Firstly, based on the literature and genotype data in HapMap, we selected 15 SNPs for the five genes. But the SNPs in these genes are far more than the selected ones. It might be necessary to study more polymorphisms in the future. Moreover, to ensure a power of not less than 0.80 with the current sample size, we selected SNPs with minor allele frequency more than 0.14, so that we could not get information about the potential variants with low allele frequency. Further large-scaled studies should detect more SNPs of the genes with lower allele frequency.

In conclusion, we identified gene-gene interaction of INSIG-SCAP-SREBP pathway on risk of obesity, revealing that these genes affect obesity more likely through a complex interaction pattern than single gene effect. Our findings await further studies in other ethnic population and functional studies to elucidate the biological mechanism.

## Supplementary Material

Supplementary Table 1: Genotyping information of the 15 SNPs in INSIG-SCAP-SREBP pathway.
Supplementary Table 2: Results of logistic regression for the 3-locus (rs9308762/rs12487736/rs1883205) model from GMDR analyses.

## Figures and Tables

**Table 1:** General characteristics of the study groups.

	Obese	Nonobese	*P* value
Number	705	1325	—
Age (year)	12.85 ± 2.59	12.93 ± 2.72	0.500
Male (%)	484 (68.7)	734 (55.4)	<0.001
Body mass index (BMI)	28.12 ± 3.94	21.53 ± 3.45	<0.001

Values were provided as mean ± SD if not indicated otherwise.

**Table 2:** Association of the 15 SNPs in INSIG-SCAP-SREBP pathway with obesity.

	Nearest gene	Effect allele	Obese vs nonobese			
			OR	95% CI		Nominal *P* value^a^
rs2721	*INSIG1 *	T	0.94	0.81	1.09	0.394
rs13223383	*INSIG1 *	T	0.90	0.78	1.04	0.164
rs7566605	INSIG2	C	1.04	0.91	1.20	0.547
rs10185316	*INSIG2 *	G	1.04	0.87	1.25	0.665
rs13428113	INSIG2	C	0.98	0.86	1.11	0.714
rs17047757	*INSIG2 *	G	1.04	0.89	1.21	0.622
rs9308762	*INSIG2 *	T	1.03	0.90	1.17	0.710
rs12487736	*SCAP *	G	1.15	1.01	1.32	0.039
rs12490383	*SCAP *	C	1.17	1.02	1.33	0.026
rs2297508	*SREBP1 *	C	1.02	0.85	1.23	0.813
rs60282872	*SREBP1 *	DEL	0.88	0.67	1.16	0.367
rs7287010	*SREBP2 *	T	1.03	0.89	1.18	0.706
rs1883205	*SREBP2 *	T	0.92	0.80	1.05	0.222
rs133290	*SREBP2 *	C	0.98	0.85	1.14	0.825
rs2228314	*SREBP2 *	C	1.17	1.00	1.37	0.051

^a^Adjusted for sex, age, age square, and study population.

*INSIG1*, insulin induced gene 1; *INSIG2*, insulin induced gene 2; *SCAP*, SREBP cleavage-activating protein gene; *SREBP1,* sterol regulatory element binding protein 1 gene; *SREBP2*, sterol regulatory element binding protein 2 gene.

**Table 3:** Frequency differences of 9 high-risk genotype combinations of INSIG-SCAP-SREBP pathway between obese and nonobese children.

rs9308672/rs12487736/rs1883205	Obese (*n* = 693)		Nonobese (*n* = 1311)		Percentage ratio
	Number	Percentage	Number	Percentage	(obese/nonobese)
CC/GG/CC	15	2.16	23	1.75	1.23
CC/AG/CT	44	6.35	63	4.81	1.32
CC/GG/TT	8	1.15	10	0.76	1.51
CT/AA/CC	39	5.63	42	3.20	1.76
CT/AG/CC	55	7.94	90	6.86	1.16
CT/GG/CC	37	5.34	45	3.43	1.56
CT/AG/TT	37	5.34	55	4.20	1.27
TT/GG/CT	37	5.34	37	2.82	1.89
TT/GG/TT	13	1.88	10	0.76	2.46

Total	285	41.13	375	28.60	1.44

## References

[1] Raymond SU, Leeder S, Greenberg HM (2006). Obesity and cardiovascular disease in developing countries: a growing problem and an economic threat. *Current Opinion in Clinical Nutrition & Metabolic Care*.

[2] Prentice AM (2006). The emerging epidemic of obesity in developing countries. *International Journal of Epidemiology*.

[3] Wang Y, Lim H (2012). The global childhood obesity epidemic and the association between socio-economic status and childhood obesity. *International Review of Psychiatry*.

[4] Brown MS, Goldstein JL (1999). A proteolytic pathway that controls the cholesterol content of membranes, cells, and blood. *Proceedings of the National Academy of Sciences of the United States of America*.

[5] Hua X, Nohturfft A, Goldstein JL, Brown MS (1996). Sterol resistance in CHO cells traced to point mutation in SREBP cleavage-activating protein. *Cell*.

[6] Sakai J, Nohturfft A, Cheng D, Ho YK, Brown MS, Goldstein JL (1997). Identification of complexes between the COOH-terminal domains of sterol regulatory element-binding proteins (SREBPS) and SREBP cleavage-activating protein. *The Journal of Biological Chemistry*.

[7] Liu X, Li Y, Lu X (2010). Interactions among genetic variants from SREBP2 activating-related pathway on risk of coronary heart disease in Chinese Han population. *Atherosclerosis*.

[8] Yang T, Espenshade PJ, Wright ME (2002). Crucial step in cholesterol homeostasis: sterols promote binding of SCAP to INSIG-1, a membrane protein that facilitates retention of SREBPs in ER. *Cell*.

[9] Yabe D, Brown MS, Goldstein JL (2002). Insig-2, a second endoplasmic reticulum protein that binds SCAP and blocks export of sterol regulatory element-binding proteins. *Proceedings of the National Academy of Sciences of the United States of America*.

[10] Brown AJ, Sun L, Feramisco JD, Brown MS, Goldstein JL (2002). Cholesterol addition to ER membranes alters conformation of SCAP, the SREBP escort protein that regulates cholesterol metabolism. *Molecular Cell*.

[11] Herbert A, Gerry NP, McQueen MB (2006). A common genetic variant is associated with adult and childhood obesity. *Science*.

[12] Hall DH, Rahman T, Avery PJ, Keavney B (2006). INSIG-2 promoter polymorphism and obesity related phenotypes: association study in 1428 members of 248 families. *BMC Medical Genetics*.

[13] Kumar J, Sunkishala RR, Karthikeyan G, Sengupta S (2007). The common genetic variant upstream of *INSIG2* gene is not associated with obesity in Indian population. *Clinical Genetics*.

[14] Lyon HN, Emilsson V, Hinney A (2007). The association of a SNP upstream of *INSIG2* with body mass index is reproduced in several but not all cohorts. *PLoS Genetics*.

[15] Smith AJ, Cooper JA, Li LK, Humphries SE (2007). *INSIG2* gene polymorphism is not associated with obesity in Caucasian, Afro-Caribbean and Indian subjects. *International Journal of Obesity*.

[16] Wang HJ, Zhang H, Zhang SW, Pan YP, Ma J (2008). Association of the common genetic variant upstream of *INSIG2* gene with obesity related phenotypes in Chinese children and adolescents. *Biomedical and Environmental Sciences*.

[17] Deka R, Xu L, Pal P (2009). A tagging SNP in *INSIG2* is associated with obesity-related phenotypes among Samoans. *BMC Medical Genetics*.

[18] Fornage M, Papanicolaou G, Lewis CE, Boerwinkle E, Siscovick DS (2010). Common *INSIG2* polymorphisms are associated with age-related changes in body size and high-density lipoprotein cholesterol from young adulthood to middle age. *Metabolism: Clinical and Experimental*.

[19] Le Hellard S, Theisen FM, Haberhausen M (2009). Association between the insulin-induced gene 2 (*INSIG2*) and weight gain in a German sample of antipsychotic-treated schizophrenic patients: perturbation of SREBP-controlled lipogenesis in drug-related metabolic adverse effects?. *Molecular Psychiatry*.

[20] Smith EM, Zhang Y, Baye TM (2010). *INSIG1* influences obesity-related hypertriglyceridemia in humans. *The Journal of Lipid Research*.

[21] Fiegenbaum M, Silveira FR, van der Sand CR (2005). Determinants of variable response to simvastatin treatment: the role of common variants of *SCAP, SREBF-1a* and *SREBF-2* genes. *The Pharmacogenomics Journal*.

[22] Vedie B, Jeunemaitre X, Megnien JL, Atger V, Simon A, Moatti N (2001). A new DNA polymorphism in the 5′ untranslated region of the human *SREBP-1a* is related to development of atherosclerosis in high cardiovascular risk population. *Atherosclerosis*.

[23] Salek L, Lutucuta S, Ballantyne CM, Gotto AM, Marian AJ (2002). Effects of *SREBF-1a* and *SCAP* polymorphisms on plasma levels of lipids, severity, progression and regression of coronary atherosclerosis and response to therapy with fluvastatin. *Journal of Molecular Medicine*.

[24] Yaju D, Ruixing Y, Yiyang L (2009). Polymorphism of the sterol regulatory element-binding protein-2 gene and its association with serum lipid levels in the Guangxi Hei Yi Zhuang and Han populations. *The American Journal of the Medical Sciences*.

[25] Liu JX, Liu J, Guo Q, Liu J (2012). Association of sterol regulatory element binding protein-1c genetic polymorphisms rs2297508 and rs11868035 with type 2 diabetes mellitus in Gansu Han and Dongxiang population. *Zhonghua Yi Xue Yi Chuan Xue Za Zhi*.

[26] Eberlé D, Clément K, Meyre D (2004). *SREBF-1* gene polymorphisms are associated with obesity and type 2 diabetes in French obese and diabetic cohorts. *Diabetes*.

[27] Grarup N, Stender-Petersen KL, Andersson EA (2008). Association of variants in the sterol regulatory element-binding factor 1 (*SREBF1*) gene with type 2 diabetes, glycemia, and insulin resistance a study of 15,734 Danish subjects. *Diabetes*.

[28] Zavattari P, Loche A, Civolani P (2010). An *INSIG2* polymorphism affects glucose homeostasis in Sardinian obese children and adolescents. *Annals of Human Genetics*.

[29] Reinehr T, Hinney A, Toschke AM, Hebebrand J (2009). Aggravating effect of *INSIG2* and *FTO* on overweight reduction in a one-year lifestyle intervention. *Archives of Disease in Childhood*.

[30] Wang D, Ma J, Zhang S (2010). Association of the *MC4R* V103I polymorphism with obesity: a Chinese case-control study and meta-analysis in 55,195 individuals. *Obesity*.

[31] Ji CY (2005). Report on childhood obesity in China (1)—body mass index reference for screening overweight and obesity in Chinese school-age children. *Biomedical and Environmental Sciences*.

[32] Wang HJ, Zhang H, Zhang J, Wang Y, Ma J (2010). Association of abnormal lipid metabolism with *INSIG2* gene variant in overweight and obese children. *Zhonghua Liu Xing Bing Xue Za Zhi*.

[33] Gao X (2011). Multiple testing corrections for imputed SNPs. *Genetic Epidemiology*.

[34] Lou XY, Chen GB, Yan L (2007). A generalized combinatorial approach for detecting gene-by-gene and gene-by-environment interactions with application to nicotine dependence. *The American Journal of Human Genetics*.

[35] Purcell S, Neale B, Todd-Brown K (2007). PLINK: a tool set for whole-genome association and population-based linkage analyses. *The American Journal of Human Genetics*.

[36] Mei H, Cuccaro ML, Martin ER (2007). Multifactor dimensionality reduction-phenomics: a novel method to capture genetic heterogeneity with use of phenotypic variables. *The American Journal of Human Genetics*.

[37] Chen Q, Yu CQ, Tang X (2011). Interactions of renin-angiotensin system gene polymorphisms and antihypertensive effect of benazepril in Chinese population. *Pharmacogenomics*.

[38] Shimano H (2009). SREBPs: physiology and pathophysiology of the SREBP family. *The FEBS Journal*.

[39] Hartwell L (2004). Robust interactions. *Science*.

[40] Phillips PC (2008). Epistasis—the essential role of gene interactions in the structure and evolution of genetic systems. *Nature Reviews Genetics*.

[41] Liou YJ, Bai YM, Lin E (2012). Gene-gene interactions of the *INSIG1* and *INSIG2* in metabolic syndrome in schizophrenic patients treated with atypical antipsychotics. *The Pharmacogenomics Journal*.

[42] Talbert ME, Langefeld CD, Ziegler JT, Haffner SM, Norris JM, Bowden DW (2009). *INSIG2* SNPs associated with obesity and glucose homeostasis traits in hispanics: the IRAS family study. *Obesity*.

[43] Baylin A, Deka R, Tuitele J, Viali S, Weeks DE, Mcgarvey ST (2013). *INSIG2* variants, dietary patterns and metabolic risk in Samoa. *European Journal of Clinical Nutrition*.

[44] Okada Y, Kubo M, Ohmiya H (2012). Common variants at *CDKAL1* and *KLF9* are associated with body mass index in east Asian populations. *Nature Genetics*.

