# Supplementary material for: The Gene-Gene Interaction of INSIG-SCAP-SREBP Pathway on the Risk of Obesity in Chinese Children
Source: Biomed Res Int. 2014 Jun 17;2014:538564. doi: 10.1155/2014/538564 (PMC4083216; doi:10.1155/2014/538564)
Supplement: Supplementary file 1 — Supplementary Table 1: Genotyping information of the 15 SNPs in INSIG-SCAP-SREBP pathway. Supplementary Table 2: Results of logistic regression for the 3-locus (rs9308762/rs12487736/rs1883205) model from GMDR analyses. [file 538564.f1.pdf]

Supplementary table 1 Genotyping information of the 15 SNPs in INSIG-SCAP-SREBP pathway

					Allele						Genotype（11/12/22）	
					Effect	Other					Non-obese	Obese
	Nearest gene	Chr.	Position [a]	Source [b]	(1)	(2)	Call rate	Total sample（11/12/22）	EAF	HWE *P*-value	group	group
rs2721	*INSIG1*	7	154718676	reference[20]	T	G	99.95%	189/839/1001	0.300	0.491	135/538/651	54/301/350
rs13223383	*INSIG1*	7	154734715	reference[19]	T	G	99.75%	185/874/966	0.307	0.527	126/575/622	59/299/344
rs7566605	INSIG2	2	118552495	reference[11]	C	G	100.00%	283/901/846	0.361	0.084	181/590/554	102/311/292
rs10185316	*INSIG2*	2	118560948	reference[18]	G	C	99.85%	53/497/1477	0.149	0.153	32/327/963	21/170/514
rs13428113	INSIG2	2	118563355	tagSNP	C	T	100.00%	513/977/540	0.493	0.093	336/641/348	177/336/192
rs17047757	*INSIG2*	2	118577905	tagSNP	G	A	99.90%	140/765/1123	0.258	0.532	90/492/741	50/273/382
rs9308762	INSIG2	2	118580344	reference[17,33,34]	T	C	100.00%	567/989/474	0.523	0.288	370/646/309	197/343/165
rs12487736	*SCAP*	3	47434683	reference[21]	G	A	99.30%	556/1003/457	0.525	0.909	347/662/309	209/341/148
rs12490383	*SCAP*	3	47469161	reference [19]	C	T	99.56%	471/1013/537	0.484	0.873	289/667/364	182/346/173
rs2297508	*SREBP1*	17	17656042	reference [25, 27]	C	G	99.95%	43/531/1455	0.152	0.501	31/339/954	12/192/501
rs60282872	*SREBP1*	17	17680892	reference[22, 23]	DEL	C	99.80%	13/235/1778	0.064	0.09	12/154/1156	1/81/622
rs7287010	*SREBP2*	22	40564883	tagSNP	T	C	99.85%	280/975/772	0.379	0.317	183/634/506	97/341/266
rs1883205	*SREBP2*	22	40572966	tagSNP	T	C	99.90%	356/1007/665	0.424	0.452	232/673/418	124/334/247
rs133290	*SREBP2*	22	40597551	tagSNP	C	A	99.90%	204/886/938	0.319	0.805	137/580/607	67/306/331
rs2228314	*SREBP2*	22	40606688	reference [7, 24]	C	G	99.85%	101/722/1204	0.228	0.587	64/447/812	37/275/392

[a] Positions according to Build 36, NCBI (http://www.ncbi.nlm.nih.gov/snp/).

[b] The NO. of the reference is according to the reference list.

Abbreviations: *INSIG1*, insulin induced gene 1; *INSIG2*, insulin induced gene 2; *SCAP*, SREBP cleavage-activating protein gene; *SREBP1*, sterol regulatory element binding protein 1 gene; *SREBP2*, sterol regulatory element binding protein 2 gene; Chr., Chromosome; EAF, effect allele frequency; HWE, Hardy Weinberg Equilibrium.

带格式的：宽度： 29.7 厘米，高度： 21 厘米

带格式表格

Supplementary table 2. Results of logistic regression for the 3-locus (rs9308762/rs12487736/rs1883205) model from GMDR analyses

	N	Non-obese(%)	Obese(%)	OR	95% CI	*P*-value
Low-risk genotypes of rs9308762/rs12487736/rs1883205						
CC/AA/CC	43	28(65.1)	15(34.9)	reference		
CC/AA/CT	66	47(71.2)	19(28.8)	0.755	0.331-1.718	0.502
CC/AA/TT	26	19(73.1)	7(26.9)	0.688	0.236-2.004	0.493
CC/AG/CC	64	43(67.2)	21(32.8)	0.912	0.403-2.061	0.824
CC/AG/TT	43	30(69.8)	13(30.2)	0.809	0.328-1.997	0.646
CC/GG/CT	65	45(69.2)	20(30.8)	0.830	0.366-1.882	0.655
CT/AA/CT	93	70(75.3)	23(24.7)	0.613	0.280-1.344	0.222
CT/AA/TT	33	23(69.7)	10(30.3)	0.812	0.307-2.145	0.674
CT/AG/CT	266	184(69.2)	82(30.8)	0.832	0.422-1.640	0.595
CT/GG/CT	146	100(68.5)	46(31.5)	0.859	0.419-1.760	0.677
CT/GG/TT	44	31(70.5)	13(29.5)	0.783	0.318-1.928	0.594
TT/AA/CC	33	24(72.7)	9(27.3)	0.700	0.260-1.884	0.480
TT/AA/CT	62	41(66.1)	21(33.9)	0.956	0.422-2.168	0.914
TT/AA/TT	20	15(75.0)	5(25.0)	0.622	0.189-2.047	0.435
TT/AG/CC	110	75(68.2)	35(31.8)	0.871	0.414-1.834	0.716
TT/AG/CT	119	83(69.7)	36(30.3)	0.810	0.387-1.695	0.575
TT/AG/TT	57	39(68.4)	18(31.6)	0.862	0.372-1.995	0.728
TT/GG/CC	65	45(69.2)	20(30.8)	0.830	0.366-1.882	0.655
High-risk genotypes of rs9308762/rs12487736/rs1883205						
CC/AG/CT	107	63(58.9)	44(41.1)	1.304	0.625-2.721	0.480
CC/GG/CC	38	23(60.5)	15(39.5)	1.217	0.493-3.005	0.670
CC/GG/TT	18	10(55.6)	8(44.4)	1.493	0.487-4.583	0.483
CT/AA/CC	81	42(51.9)	39(48.1)	1.733	0.808-3.720	0.158
CT/AG/CC	145	90(62.1)	55(37.9)	1.141	0.560-2.323	0.717
CT/AG/TT	92	55(59.8)	37(40.2)	1.256	0.591-2.666	0.553
CT/GG/CC	82	45(54.9)	37(45.1)	1.535	0.715-3.292	0.271
TT/GG/CT	74	37(50.0)	37(50.0)	1.867	0.860-4.053	0.115
TT/GG/TT	23	10(43.5)	13(56.5)	2.427	0.861-6.837	0.093
Genotype combination						
Low-risk group	1355	942(69.5)	413(30.5)	reference		
High-risk group	660	375(56.8)	285(43.2)	1.799	1.475-2.193	6.61×10^{-9}
